# Consumption of dairy products in relation to the presence of clinical knee osteoarthritis: The Maastricht Study

**DOI:** 10.1007/s00394-018-1818-7

**Published:** 2018-09-21

**Authors:** Karlijn F. M. Denissen, Annelies Boonen, Johannes T. H. Nielen, Anouk L. Feitsma, Ellen G. H. M. van den Heuvel, Pieter J. Emans, Coen D. A. Stehouwer, Simone J. S. Sep, Martien C. J. M. van Dongen, Pieter C. Dagnelie, Simone J. P. M. Eussen

**Affiliations:** 1 Department of Epidemiology, Maastricht University, PO Box 616, 6200 MD Maastricht, The Netherlands; 2 CAPHRI Care and Public Health Research Institute, Maastricht University, PO Box 616, 6200 MD Maastricht, The Netherlands; 3 Division of Rheumatology, Department of Internal Medicine, Maastricht University Medical Center +, PO Box 5800, 6202 AZ Maastricht, The Netherlands; 4 Department of Clinical Pharmacy and Toxicology, Maastricht University Medical Center +, PO Box 5800, 6202 AZ Maastricht, The Netherlands; 5 FrieslandCampina, Stationsplein 4, PO Box 1551, 3800 BN Amersfoort, The Netherlands; 6 Department of Orthopaedics, Maastricht University Medical Center +, PO Box 5800, 6202 AZ Maastricht, The Netherlands; 7 CARIM School for Cardiovascular Diseases, Maastricht University, PO Box 616, 6200 MD Maastricht, The Netherlands; 8 Department of Rehabilitation Medicine, Maastricht University, PO Box 616, 6200 MD Maastricht, The Netherlands

**Keywords:** Dairy products, FFQ, Knee osteoarthritis, Milk, Observational studies, Osteoarthritis

## Abstract

**Purpose:**

Observational studies showed inverse associations between milk consumption and knee osteoarthritis (knee OA). There is lack of information on the role of specific dairy product categories. We explored the association between dairy consumption and the presence of knee osteoarthritis in 3010 individuals aged 40–75 years participating in The Maastricht Study.

**Methods:**

The presence of knee OA was defined according to a slightly modified version of the American College of Rheumatology (ACR) clinical classification criteria. Data on dairy consumption were appraised by a 253-item FFQ covering 47 dairy products with categorization on fat content, fermentation or dairy type. Multivariable logistic regression analyses were performed to estimate odd ratios (ORs) and 95% confidence intervals (95%CI), while correcting for relevant factors.

**Results:**

427 (14%) participants were classified as having knee OA. Significant inverse associations were observed between the presence of knee OA and intake of full-fat dairy and Dutch, primarily semi-hard, cheese, with OR for the highest compared to the lowest tertile of intake of 0.68 (95%CI 0.50–0.92) for full-fat dairy, and 0.75 (95%CI 0.56–0.99) for Dutch cheese. No significant associations were found for other dairy product categories.

**Conclusion:**

In this Dutch population, higher intake of full-fat dairy and Dutch cheese, but not milk, was cross-sectionally associated with the lower presence of knee OA. Prospective studies need to assess the relationship between dairy consumption, and in particular semi-hard cheeses, with incident knee OA.

## Introduction

Osteoarthritis (OA) is one of the most prevalent musculoskeletal disorders affecting the lives of many older adults worldwide [1]. With pain as predominant symptom, hip and knee OA were ranked as the 11th leading cause of global disability in 2010 [2]. OA is associated with a high economic burden due to, e.g. costs of care, productivity loss, or comorbid diseases [3]. Epidemiological studies have consistently shown that increasing age, female sex, obesity, a previous knee injury, and repetitive mechanical forces on the knee during occupation are strong risk factors for developing knee OA [4, 5]. In view of the ageing population and the rapidly increasing incidence of OA [6], it is of major public health relevance to identify modifiable lifestyle factors that can prevent or delay progression of this disease [7].

Disturbances of different metabolic pathways within the tissues of the osteoarthritic joint are increasingly recognised as a key feature of the pathophysiology of OA [8]. Although it is evident that such pathways may be influenced by dietary factors [9], research on the role of nutrition in OA etiology is relatively scarce and mainly focused on the antioxidant vitamins A, C and E, and vitamin D, all of which were negatively associated with knee OA progression [10–12]. Given the complex interplay between nutrients in the diet [13], another approach is to focus on the effect of specific foods or food groups of interest. Dairy products are particularly relevant as they are excellent sources of vitamins, minerals and proteins, which have long been recognised for their crucial role in bone [14, 15] and skeletal muscle health [16]. Given the close ties between bone, skeletal muscle and joint functions in movements, dairy products are most likely to play a role also in joint health, e.g. knee OA.

In two first studies, clear associations between milk consumption and knee OA were found [17, 18]. A cross-sectional study among 655 Turkish individuals [17] showed that the odds of radiologically and clinical diagnosed knee OA was three times lower in daily milk consumers compared to infrequent consumers (OR 0.29, 95% CI 0.13–0.65) [17]. No significant associations were found for cheese or yoghurt. While the observed relation with milk intake seems impressive, the study by Kaçar and coworkers did not correct for potentially important confounding factors [17].

In a prospective study of 2481 Americans participating in the Osteoarthritis Initiative [18], milk consumption in women was associated with reduced 4-year progression of knee OA, i.e. a 24–32% reduction in decrease of radiologically assessed joint space width (*P*_trend_ = 0.014), whereas an increased progression was observed for the women with the highest cheese consumption compared to no cheese consumption (*P* = 0.003). No significant associations were found for yoghurt or total dairy intake [18].

Although these two reports suggest a potential negative association between some types of dairy and osteoarthritis risk, there are still major knowledge gaps. First, substantial differences between countries exist in type and quantity of dairy consumed, which may result in different associations between dairy intake and knee OA across countries. For example, total per capita milk consumption in the US is four times higher than in Turkey [19], and whereas non-fat or low-fat dairy constitutes the highest proportion of dairy consumed in the US [20], dairy consumed in Turkey is often full-fat. Furthermore, Turkey has the 2nd highest global yoghurt consumption per capita [21], whereas yoghurt consumption in the United States (US) is among the lowest in the world [21]. Also between European countries, marked disparities in habitual dairy consumption have been observed [22]. Second, and most importantly, no information is available on the differential effect of specific dairy product categories such as fermented dairy, non-fermented dairy, low-fat or full-fat dairy.

Based on these previous studies, we hypothesize that higher consumption of dairy products is associated with a lower odds of having clinical knee OA. In The Maastricht Study, a Dutch population-based study, cross-sectional data have been collected on intake of nearly 50 dairy products, other dietary factors, symptoms and signs of knee OA and known determinants of knee OA. Such extensive data collection offers a unique opportunity to explore the potential differential role of specific dairy product categories. Findings could serve as a starting point for future experimental research or lab studies and, therefore, contribute to unravelling causal relationships between dietary factors and knee OA. This could result in new treatment options, such as new products, a targeted dietary advice, or even preventive strategies for knee OA. In the present report, we, therefore, explored the cross-sectional association between dairy product intake and clinical knee OA in The Maastricht Study.

## Methods

### Study design and population

Data from The Maastricht Study, an ongoing observational prospective population-based cohort study, were used. Its rationale and methodology have been described previously [23]. In brief, the study focuses on the aetiology, pathophysiology, classic complications, and emerging comorbidities of type 2 diabetes (T2DM), and is characterized by an extensive phenotyping approach. Eligible participants were individuals between 40 and 75 years of age and living in the southern part of the Netherlands. Participants were recruited through mass media campaigns and from the municipal registries and the regional Diabetes Patient Registry via mailings. For reasons of efficiency, recruitment was stratified according to known T2DM status, with an oversampling of individuals with T2DM.

This report includes cross-sectional data from 3451 participants who completed the baseline survey between November 2010 and September 2013. The examinations of each participant were performed within a time frame of 3 months. The study was approved by the institutional medical ethical committee (NL31329.068.10) and the Minister of Health, Welfare, and Sports of the Netherlands (Permit 131088-105234-PG). All participants gave written informed consent.

From the 3451 participants who completed the baseline survey, we excluded participants that did not fill out the food frequency questionnaire (FFQ) (*n* = 163), had implausible energy intake, i.e. < 800 or > 4200 kcal/day for men and < 500 or > 3500 kcal/day for women (*n* = 65) [24], had another type of diabetes than T2DM (*n* = 35), did not participate in examinations for knee OA (*n* = 106), or had no data on the presence of knee pain (*n* = 72), thereby including 3010 participants in current analyses. The 441 participants excluded from this study, were slightly younger (58.2 vs 60.0 years) and more frequently male (56.9 vs 50.6%) than participants included in the analyses. They also had lower educational attainment (low: 37.2 vs 32.9%; medium: 28.8 vs 28.0%; high level: 30.8 vs 39.1%), but did not differ with respect to BMI compared to participants in the present analyses.

### Clinical knee osteoarthritis

Measurements on emerging comorbidities of T2DM within The Maastricht Study included a disease-specific questionnaire and physical examination on symptoms and signs of knee OA [23]. All measurements were performed by trained research assistants using standardized protocols.

A participant was classified as having clinical knee OA (hereinafter referred to as knee OA) when one or two knees fulfilled the traditional American College of Rheumatology (ACR) clinical classification criteria for OA of the knee [25]. According to this definition, knee OA was considered to be present if the participant experienced recurrent non-traumatic knee pain for at least 4 weeks during the previous 6 months, and showed at least three out of the following clinical signs: (a) age > 50 years, (b) start-up pain or stiffness < 30 min of duration, (c) sensitivity of the bony margins of the joint upon palpation, (d) bony enlargement as assessed on physical examination, (e) lack of palpable warmth of the synovium, or (f) crepitus, i.e. a crunching or popping sound in the knee joint on active motion. In current analyses, we used a slightly modified version of knee OA which did not include the clinical sign crepitus, because this was not measured within the present study. While excluding the criterium ‘crepitus’ from the denominator, the number of criteria (*n* = 3) that needed to be fulfilled to classified as a case remained identical. As such, we used even a slightly stricter definition of knee OA. Since end-stage primary knee OA is the indication for 88–96% of all knee-replacement surgeries [26, 27], participants with a knee replacement (*n* = 182) were defined as having knee OA as well.

### Dietary intake

Habitual dietary intake was estimated with a tailor-made FFQ which our research group developed using the Dutch National FFQTOOL^®^ [28]. This FFQ comprises 253 food items covering 23 product groups, such as fruit, vegetables, fish, meat and dairy products, and assesses the average frequency and quantity consumed during the past 12 months. Dairy product intake was appraised by 47 items covering unflavoured milk (three items), milk-based drinks (chocolate milk, one item; breakfast drink, one item), buttermilk (one item), custard and pudding (six items), cheese (nine items), yoghurt (ten items), quark (seven items), dairy with probiotics (two items, including both milk- and yoghurt-based variants), butter (three items), evaporated milk (three items), and ready-to-eat porridge (one item). Moreover, as pointed out in the introduction, this extensive information on dairy food items consumed, enabled to make a distinction between full-fat, semi-skimmed and skimmed products, as well as fermented and non-fermented products to explore the differential effect of the major dairy product categories [29] (Table 1). For the present report, dairy consumption was expressed as servings per day (servings/day) [30]. The customary serving sizes for evaporated milk (8 g) and butter (6 g) were not used because this would result in an overestimation of dairy consumption in consumers of these products. Accordingly, 20 g of cheese or 150 g of all other dairy products counted as 1 serving per day. FFQ-based intake of energy and micronutrients was calculated using the Dutch Food Composition Database (NEVO), version 2011 [31].

**Table 1:** Categorization of 47 dairy food items into specific categories

Dairy product	Composition
Total	All dairy products mentioned below
Full-fat dairy	Full-fat unflavoured milk or flavoured milk-based drinks, unflavoured or fruit flavoured yoghurt and quark, custard or pudding, full-fat Dutch and foreign cheese, evaporated milk, butter, and ready-to-eat porridge
Semi-skimmed dairy	Semi-skimmed unflavoured milk or flavoured milk-based drinks, unflavoured or fruit flavoured yoghurt and quark, custard and drinking yoghurt
Skimmed dairy	Skimmed unflavoured milk or flavoured milk-based drinks, unflavoured or fruit flavoured yoghurt and quark, drinking yoghurt, buttermilk and custard, low-fat Dutch and foreign cheese, and evaporated milk
Fermented dairy	Yoghurt: all fat percentages, unflavoured or fruit flavoured, spoonable or drinking yoghurt
	Cheese: all fat percentages, both Dutch and foreign, also including spreadable cheese
	Quark: all fat percentages, unflavoured or fruit flavoured
	Butter milk
Non-fermented dairy	Unflavoured milk: all fat percentages, sweetened with sugar or artificial sweeteners
	Custard and pudding: all fat percentages, sugar sweetened
	Ready-to-eat porridge
Unflavoured milk	Unflavoured milk: all fat percentages
Cheese, total	Dutch cheese: semi-hard full-fat, semi-hard low-fat, and spreadable cheese of all fat percentages
	Foreign cheese: full-fat and low-fat from soft to hard cheese (all moisture contents), and spreadable cheese of all fat percentages
Cheese, Dutch	Dutch cheese: semi-hard (e.g. Gouda, Maasdam, Edam) full-fat, semi-hard low-fat, and spreadable cheese of all fat percentages
Yoghurt, total	Unflavoured yoghurt: all fat percentages
	Fruit flavoured yoghurt: all fat-percentages, sweetened with sugar or artificial sweeteners
	Drinking yoghurt: skimmed and semi-skimmed, sweetened with sugar or artificial sweeteners
Quark, total	Unflavoured quark: all fat percentages
	Fruit flavoured quark: al fat percentages, sweetened with sugar or artificial sweeteners

### Covariates

During physical examination, body weight and height were measured to the nearest 0.5 kg and 0.1 cm, respectively [23]; subsequently, body mass index (BMI) was calculated as weight (kg)/height^2^ (m). Energy intake (kcal/day), alcohol intake [none, low (men: ≤ 14 units, women: ≤7 units/week), high (men: > 14 units, women: > 7 units/week)], and intake of meat, fish and shellfish, vegetables and fruits (g/day) were derived from the FFQ. Information on age and sex of the participants was extracted from study files; smoking status (never, current, or former smoker) and level of highest educational attainment (low, middle, or high level) were based on self-report. The participants’ history of sports-related knee injury (yes, no) and present or past occupational exposure to knee loading (no regular kneeling/squatting, regular kneeling/squatting with or without lifting objects > 5 kg) were derived from a brief interview preceding the clinical examination for knee OA. Because only a small proportion of the participants had to squat/kneel without having to lift, these individuals were clustered into one category with participants that both had to squat/kneel and lift. Diabetes status was defined using the World Health Organization 2006 criteria for glucose metabolism [32] and categorized as no T2DM (including individuals with normal glucose tolerance, impaired fasting glucose and impaired glucose tolerance), and T2DM. Detailed information on these measurements is provided elsewhere [23].

### Statistical analyses

Data analysis was performed using the software package SPSS Statistics version 23.0 for Windows (SPSS, IBM Corp., Armonk, NY, USA). The characteristics of study participants are reported as mean and standard deviation (SD) for continuous variables and as proportions (%) for categorical variables.

Multivariable logistic regression analyses were performed to determine the association between each category of dairy product consumed (presented in Table 1), and the presence of knee OA relative to no presence of knee pain. Results are presented as odds ratios (OR) with corresponding 95% confidence intervals (95% CI), and a two-sided *p* value of < 0.05 was considered statistically significant.

Dairy product consumption was expressed as tertiles, based on the distribution in the group of participants without knee pain, with the first tertile assigned as reference group. Due to low consumption, unflavoured milk had a first tertile cut-off point equal to zero. For this dairy product category, non-consumers served as reference group and were compared with participants having an intake below or above the median intake of the consumers. When testing for linear trend across tertiles or groups of dairy product consumption, categorical variables were entered as a continuous variable as well. Logistic regression models were computed as “crude models”, adjusted for age (continuous), sex and diabetes status (no T2DM, T2DM) only (model 1), or as fully adjusted models (model 2) which were additionally adjusted for the intake of energy (kcal), meat, fish and shellfish, vegetables and fruits (g/day) to represent overall diet, and for known determinants of knee OA that, in our dataset, were significantly associated with knee OA in univariate analysis, i.e. education (low, middle, high), smoking (never, former, current), BMI (continuous), history of sports-related knee injury (yes, no), occupational exposure to knee loading (no squatting/kneeling, yes squatting/kneeling), and alcohol consumption (none, low, high). The intake of vitamin A, C, D, and E was not associated with knee OA. Explorative analyses showed that type and duration of current non-occupational physical activity was possibly influenced by the presence of knee OA, e.g. participants with knee OA were less likely to practice sports with a moderate to high level of (knee) joint loading, indicating reverse causality. Therefore, current non-occupational physical activity was not included as potential confounder. All relevant covariates described above were simultaneously added to the regression models.

In secondary analyses, we evaluated effect modification; first by sex, based on the observation that in the Osteoarthritis Initiative milk consumption was protective for knee OA progression in women only [18]; second by BMI and occupational exposure to knee loading, because it is conceivable that the relation between dairy consumption and knee OA differs depending on the mechanical load on the knee; and last, by diabetes status, because the presence of diabetes could potentially modify the association between dairy consumption and knee OA. Interaction terms for sex, BMI, occupational exposure to knee loading, and diabetes status were alternately added to the fully adjusted regression models; *P*_interaction_ < 0.10 was considered statistically significant whereupon analyses were stratified for the covariate of interest to understand the relevance of the effect modification.

## Results

### Population characteristics

Of the 3010 participants included in this study, 427 individuals (14%) were classified as having knee OA (Table 2). Compared to participants without knee pain (*n* = 2364), individuals with knee OA were older and less frequently male, had lower educational attainment, more likely to have (had) occupational exposure to kneeling or squatting, and had higher prevalence of obesity, T2DM and history of sports-related knee injury. Besides, they tended to have a lower intake of vitamin D and a higher intake of vitamin C.

**Table 2:** Population characteristics (*n* = 3010) of The Maastricht Study

	All participants (*n* = 3010)	No knee pain (*n* = 2364)	Clinical knee osteoarthritis (*n* = 427)
Age (years)	60.0 (8.14)	59.7 (8.30)	62.5 (6.54)
Sex (% male)	50.6	51.6	41.2
Highest education (*n*, %)			
Low	32.9	30.9	44.6
Medium	28.0	27.6	26.6
High	39.1	41.1	28.8
Smoking (*n*, %)			
Never	35.4	35.6	33.5
Former	52.1	51.5	55.3
Current	12.5	12.9	11.2
BMI (kg/m^2^) (*n*, %)	27.0 (4.52)	26.7 (4.30)	29.0 (5.34)
Normal	35.9	38.0	23.0
Overweight	42.2	42.5	39.6
Obese	21.9	19.4	37.5
Type 2 diabetes (*n*, %)	27.5	25.9	37.7
History of sport-related knee injury (%)	25.9	22.8	39.1
Occupational exposure to knee loading (%)			
No squatting or kneeling	76.4	77.9	70.6
Squatting or kneeling without lifting	3.5	3.5	4.0
Squatting or kneeling with lifting	20.1	18.6	25.4
Dietary intake			
Energy (kcal/day)	2178 (603)	2182 (603)	2123 (595)
Vegetables (g/day)	181 (99.0)	180 (97.5)	185 (100)
Fruits (g/day)	195 (145)	195 (146)	207 (143)
Meat (g/day)	111 (58.8)	110 (57.5)	116 (61.0)
Fish and shellfish (g/day)	24.4 (21.9)	24.7 (22.1)	22.5 (20.6)
Alcohol use (%)	18.1	17.2	25.9
None	56.1	56.8	50.4
Low	25.3	26.0	23.7
High	18.1	17.2	25.9
Vitamin A (mcg/day)	721 (652)	719 (653)	713 (655)
Vitamin C (mg/day)	128 (61.2)	127 (60.8)	134 (62.5)
Vitamin D (mcg/day)	3.88 (1.90)	3.90 (1.90)	3.75 (1.93)
Vitamin E (mg/day)	14.4 (5.66)	14.4 (5.53)	14.2 (5.84)
Calcium (mg/day)	965 (385)	965 (383)	953 (381)
Phosphorus (mg/day)	1564 (442)	1562 (439)	1550 (454)
Magnesium (mg/day)	376 (102)	375 (100)	371 (107)
Zinc (mg/day)	11.1 (3.02)	11.1 (2.98)	11.0 (3.09)

Values are presented as mean (SD) or proportions

Normal: < 25.00 kg/m^2^, Overweight: ≥ 25.00 to < 30.00 kg/m^2^, Obese: ≥ 30.00 kg/m^2^

Alcohol use—Low: ≤ 14 units (glasses)/week for men and ≤ 7 units/week for women, High: > 14 units/week for men and > 7 units/week for women

*BMI* body mass index, *SD* standard deviation

### Consumption of dairy products

Dairy products were consumed by nearly all participants. The overall mean intake of total dairy was 2.89 (SD 1.87) servings/day (Table 3). Over 99% of the participants consumed fermented dairy, including yoghurt, cheese, quark and buttermilk, and with a mean consumption of 2.26 (SD 1.70) servings/day; this accounted for the largest proportion of total dairy consumed. Mean total cheese intake, including Dutch and foreign cheese, was 1.61 (SD 1.46) servings/day. The mean Dutch cheese intake of 1.47 (SD 1.38) serving/day shows that total cheese consumption primarily consisted of Dutch cheese. Although Dutch cheese also includes spreadable cheese (Table 1), over 90% was consumed as semi-hard cheese (data not shown). Yoghurt was used by 85%, quark by 50%, and milk by just over 50% of the participants, with the smallest proportion of milk consumers found in individuals with knee OA. Except for this difference in milk consumption and a slightly lower mean intake of full-fat dairy and a higher mean intake of skimmed dairy in individuals with knee OA, no pronounced differences were observed between participants with knee OA and those without knee pain.

**Table 3:** Consumption of dairy products (servings/day and % consumers) for the total population, participants without knee pain, and participants with clinical knee osteoarthritis in The Maastricht Study

	All participants (*n* = 3010)	No knee pain (*n* = 2364)	Clinical knee osteoarthritis^a^ (*n* = 427)
Total dairy			
% Consumers	99.9	99.9	100.0
Intake servings/day	2.89 (1.87)	2.88 (1.85)	2.89 (1.97)
Full-fat dairy			
% Consumers	98.4	98.5	98.4
Intake servings/day	0.85 (1.44)	0.88 (1.47)	0.68 (1.19)
Semi-skimmed dairy			
% Consumers	87.0	86.9	87.1
Intake servings/day	0.33 (0.70)	0.33 (0.72)	0.30 (0.68)
Skimmed dairy			
% Consumers	92.7	92.6	93.2
Intake servings/day	0.86 (1.44)	0.86 (1.45)	0.95 (1.67)
Non-fermented dairy			
% Consumers	90.5	90.3	92.5
Intake servings/day	0.35 (0.80)	0.35 (0.80)	0.34 (0.88)
Fermented dairy			
% Consumers	99.3	99.4	99.3
Intake servings/day	1.94 (1.89)	1.95 (1.88)	1.87 (1.93)
Unflavored milk			
% Consumers	54.2	54.6	50.6
Intake servings/day	0.29 (0.79)	0.29 (0.80)	0.43 (0.92)
Cheese, total			
% Consumers	97.6	97.6	98.1
Intake servings/day	1.22 (1.22)	1.24 (1.39)	1.13 (1.50)
Cheese, Dutch			
% Consumers	97.0	97.0	97.9
Intake servings/day	1.09 (1.42)	1.14 (1.42)	1.00 (1.49)
Yoghurt, total			
% Consumers	85.1	85.1	85.5
Intake servings/day	0.43 (0.72)	0.43 (0.72)	0.43 (0.70)
Quark, total			
% Consumers	49.5	50.0	49.4
Intake servings/day	0.01 (0.08)	0.01 (0.08)	0.01 (0.14)

20 g of cheese or 150 g of all other dairy products are considered as 1 serving of dairy

Values are presented as median (IQR) or proportions

Bold values indicate *P* < 0.05

*SD* standard deviation

^a^Defined as presence of clinical knee osteoarthritis according to a modified version of the traditional classification criteria of the American College of Rheumatology (Altman et al. 1986) and/or presence of an artificial knee joint. See “Methods” section

Median (IQR) quark intake of the total population was only 0.01 (0.08) servings/day (Table 3). Therefore, we considered that quark intake was too low for further meaningful analysis.

### Associations between dairy product consumption and the presence of knee OA

Crude logistic regression analyses, adjusted for age, sex and diabetes status (model 1, Table 4) showed significant inverse associations between intake of full-fat dairy and Dutch cheese with knee OA. Fully adjusted models (model 2, Table 4) remained essentially the same. Participants consuming ≥ 1.4 servings of full-fat dairy/day (T3) had significantly lower odds of having knee OA than individuals consuming < 0.5 serving/day (T1) (OR 0.68, 95% CI 0.50–0.92). Consumption of Dutch cheese was associated with significant lower odds of knee OA when comparing the group with an intake of ≥ 1.7 servings/day (T3) with the group that consumed < 0.7 serving/day (T1) (OR 0.75, 95% CI 0.56–0.99). Tests for linear trend across tertiles of dairy product consumption were significant for both full-fat dairy (*P*_trend_ = 0.01) and Dutch cheese (*P*_trend_ = 0.04). Mutual adjustments of full-fat dairy and Dutch cheese with dairy products other than the dairy product or product category of interest did not alter the associations found with knee OA. Consumption of total dairy, semi-skimmed or skimmed dairy, non-fermented dairy, milk, total cheese and yoghurt was not associated with knee OA in any of the models.

**Table 4:** Cross-sectional associations between intake of dairy products and osteoarthritis of the knee

	Tertile 1	Tertile 2	Tertile 3	*P*_trend_
	OR	OR (95% CI)	OR (95% CI)	
Total dairy	< 1.9 serving/d^a^	≥ 1.9 to < 3.3 serving/d	≥ 3.3 serving/d	
	Ca/nCa^b^: 141/785	Ca/nCa: 145/791	Ca/nCa: 141/788	
Model 1^†^	1	1.01 (0.78–1.31)	0.99 (0.77–1.29)	0.95
Model 2^‡^	1	1.00 (0.75–1.32)	1.04 (0.77–1.41)	0.80
Full-fat dairy	< 0.5 serving/d	≥ 0.5 to < 1.4 serving/d	≥ 1.4 serving/d	
	Ca/nCa: 175/795	Ca/nCa: 140/776	Ca/nCa: 112/793	
Model 1^†^	1	0.86 (0.67–1.11)	**0.68 (0.52–0.88)**	**0.00**
Model 2^‡^	1	0.88 (0.67–1.16)	**0.68 (0.50–0.92)**	**0.01**
Semi-skimmed dairy	< 0.08 serving/d	≥ 0.08 to < 0.5 serving/d	≥ 0.5 serving/d	
	Ca/nCa: 139/788	Ca/nCa: 157/791	Ca/nCa: 131/785	
Model 1^†^	1	1.16 (0.90–1.49)	0.98 (0.75–1.27)	0.89
Model 2^‡^	1	1.15 (0.88–1.52)	0.97 (0.72–1.29)	0.83
Skimmed dairy	< 0.4 serving/d	≥ 0.4 to < 1.3 serving/d	≥ 1.3 serving/d	
	Ca/nCa: 131/786	Ca/nCa: 135/793	Ca/nCa: 161/785	
Model 1^†^	1	0.98 (0.75–1.27)	1.13 (0.87–1.46)	0.35
Model 2^‡^	1	0.91 (0.68–1.20)	1.07 (0.81–1.42)	0.62
Non-fermented dairy	< 0.09 serving/d	≥ 0.09 to < 0.6 serving/d ≥ 1.3 serving/d	≥ 0.6 serving/d	
	Ca/nCa: 130/774	Ca/nCa: 153/800	Ca/nCa: 144/790	
Model 1^†^	1	1.24 (0.96–1.61)	1.15 (0.89–1.50)	0.30
Model 2^‡^	1	1.19 (0.90–1.58)	1.12 (0.84–1.50)	0.46
Fermented dairy, all	< 1.4 serving/d	≥ 1.4 to < 2.5 serving/d	≥ 2.5 serving/d	
	Ca/nCa: 155/786	Ca/nCa: 131/790	Ca/nCa: 141/788	
Model 1^†^	1	0.85 (0.65–1.10)	0.89 (0.69–1.14)	0.35
Model 2^‡^	1	0.82 (0.62–1.08)	0.95 (0.71–1.27)	0.72
Unflavoured milk	non-cons	> 0 to < 0.3 serving/d	≥ 0.3 g/day	
	Ca/nCa: 211/1047	Ca/nCa: 87/619	Ca/nCa: 129/671	
Model 1^†^	1	0.80 (0.61–1.06)	1.05 (0.83–1.35)	0.84
Model 2^‡^	1	0.78 (0.58–1.04)	1.00 (0.77–1.30)	0.82
Cheese, total	< 0.9 serving/d	≥ 0.9 to < 1.9 serving/d	≥ 1.9 serving/d	
	Ca/nCa: 152/773	Ca/nCa: 142/793	Ca/nCa: 133/798	
Model 1^†^	1	0.89 (0.69–1.15)	0.81 (0.63–1.06)	0.12
Model 2^‡^	1	0.79 (0.60–1.04)	0.84 (0.63–1.12)	0.21
Cheese, Dutch	< 0.7 serving/d	≥ 0.7 to < 1.7 serving/d	≥ 1.7 serving/d	
	Ca/nCa: 159/787	Ca/nCa: 138/739	Ca/nCa: 130/838	
Model 1^†^	1	0.91 (0.70–1.17)	**0.73 (0.57–0.95)**	**0.02**
Model 2^‡^	1	0.83 (0.63–1.09)	**0.75 (0.56–0.99)**	**0.04**
Yoghurt, total	< 0.1 serving/d	≥ 0.1 to < 0.6 serving/d	≥ 0.6 serving/d	
	Ca/nCa: 130/785	Ca/nCa: 132/705	Ca/nCa: 165/874	
Model 1^†^	1	1.17 (0.89–1.53)	1.17 (0.90–1.51)	0.25
Model 2^‡^	1	1.14 (0.85–1.52)	1.19 (0.89–1.58)	0.25

Defined as presence of clinical knee osteoarthritis according to a modified version of the traditional classification criteria of the American College of Rheumatology (Altman et al. 1986) and/or presence of an artificial knee joint. See “Methods” section

*non-cons* non-consumers, *serving/d*, servings per day *T2DM* type 2 diabetes mellitus, *BMI* body mass index

^†^Model 1, multivariate logistic regression analyses adjusted for age, diabetes status and sex; Ca/nCa = 427/2364

^‡^Model 2, additionally adjusted for BMI, education, occupational exposure to knee loading, history of sport-related knee injury, smoking, intakes of energy, alcohol, meat, fish and shellfish, fruit and vegetables; Ca/nCa = 404/2241

^a^20 g of cheese or 150 g of all other dairy products are considered as 1 serving of dairy

^b^Ca/nCa: number of cases/number of non-cases

Because Dutch cheese accounted for two-third of the full-fat dairy consumed, we performed a post hoc analysis to examine whether results changed after exclusion of Dutch cheese from the full-fat dairy category. We observed that consumption from  ≥ 0.2 to <0.4 servings/day of full-fat dairy (T2) was associated with a 38% higher odds of clinical knee OA compared to an intake of < 0.2 servings/day (T1) with an OR (95% CI) of 1.38 (1.05–1.83) in the fully adjusted model, indicating that the lower odds of knee OA was most likely caused by Dutch cheese and not the other items in the full-fat dairy category.

When exploring interactions (sex, BMI, occupational exposure to knee loading, and diabetes status), a statistically significant effect modification (*P*_interaction_ < 0.10) was observed for occupational exposure to knee loading only. 124 out of 642 participants (19%) with present or past occupational exposure to squatting/kneeling and lifting, were classified as having knee OA, compared to 298 out of 2127 participants (14%) without occupational knee loading. Stratified analyses revealed a significant inverse association of dairy intake with knee OA for those with present or past occupational exposure to squatting/kneeling and lifting, i.e. total dairy (*P*_interaction_ = 0.024, OR 0.73, 95% CI 0.55–0.97, *P*_trend_ = 0.029), full-fat dairy (*P*_interaction_ = 0.059, OR 0.66, 95% CI 0.49–0.88, *P*_trend_ = 0.004), fermented dairy (*P*_interaction_ = 0.047, OR 0.73, 95% CI 0.55–0.97, *P*_trend_ = 0.030), total cheese (*P*_interaction_ = 0.014, OR 0.67, 95% CI 0.50–0.88, *P*_trend_ = 0.004), and Dutch cheese (*P*_interaction_ = 0.014, OR 0.64, 95% CI 0.49–0.85, *P*_trend_ = 0.002). No significant associations were observed for individuals not exposed to occupational knee loading, i.e. total dairy (OR 1.18, 95% CI 0.98–1.42), full-fat dairy (OR 0.90, 95% CI 0.75–1.08), fermented dairy (OR 1.09, 95% CI 0.91, 1.30), total cheese (OR 1.03, 95% CI 0.86–1.22), and Dutch cheese (OR 0.96, 95% CI 0.81–1.14). Tests for interaction with sex, BMI, and diabetes status were not significant (*P*_interaction_ > 0.10; data not shown).

## Discussion

In this cross-sectional observational study, consumption of full-fat dairy and Dutch cheese was significantly inversely associated with the presence of clinical knee OA. After adjusting for determinants of knee OA and dietary factors, participants with the highest intake of the aforementioned dairy products had approximately 30% lower odds of knee OA compared to participants with the lowest intake. No significant associations were found for any of the other dairy products or dairy product categories.

As opposed to previous studies [17, 18], we did not find evidence for an inverse association of milk consumption with knee OA. In the cross-sectional study by Kaçar et al. [17], daily milk consumers had lower odds of symptomatic knee OA than individuals with less frequent milk consumption. However, as these authors did not report serving sizes and statistical analyses were virtually unadjusted for potential confounders, we cannot compare these results with our findings. When converted to the serving sizes as used in our report, data from the US Osteoarthritis Initiative [18] revealed that baseline milk consumption of ≥ 0.7 servings/day in women with radiographic knee OA was associated with a significant reduced progression of narrowing of the joint space width during 4 years of follow-up, relative to no milk consumption, whilst adjusting for relevant covariates. In men, no significant association was observed. It is important to note that in our study population, dairy intake including milk, but also cheese, was lower than observed in the general Dutch population and in other European countries [22, 33]. When compared to milk intake in the Osteoarthritis Initiative, just over half of the participants within our study population consumed milk and only 18% consumed ≥ 0.7 servings/day, compared to 83% consuming ≥ 0.7 servings/day in this US study population. It is, therefore, possible that the null-finding for milk intake in our study population may be explained by the relatively high number of non-milk-consumers and the relatively low milk consumption among consumers to detect an association. Moreover, different biochemical mechanisms may be responsible for a potential effect of dietary components in the development vs progression of knee OA. For instance, differences in associations between dietary intake and incidence opposed to progression of knee OA have been observed for anti-oxidant vitamins and vitamin D as well [11, 12]. Growing evidence suggests that risk factors for knee OA progression are distinct from those for incident knee OA knee [34]. For example, female sex, age and BMI are predictive of knee OA development, but there is strong evidence that female sex is not predictive for knee OA progression, and for BMI and age the evidence is conflicting [34].

In the Osteoarthritis Initiative, high consumption of cheese [18] was associated with increased knee OA progression. This adverse effect of cheese consumption is in contrast with the beneficial relation between Dutch cheese consumption and knee OA as observed in our study. Over 90% of the Dutch cheese consumed in our study population consisted of Dutch semi-hard cheeses, for example Gouda, Edam or Maasdam, whereas < 10% consisted of spreadable cheeses. A wide variety of cheese is consumed in the US of which cheddar and mozzarella are the most consumed [35]. In addition, different fermentation techniques [35] are used compared to the Netherlands. As nutritional characteristics of cheese depend on the cheese-making process, such as the application of heat and the specific moulds or bacteria added [36, 37], differences in nutritional characteristics of Dutch cheese relative to cheese consumed in the US may play a role. Further, recent analyses showed that vitamin K content is much lower in soft cheeses such as mozzarella and pecorino, compared to the semi-hard cheeses often consumed in the Netherlands [38]. Finally, it is important to acknowledge that the OAI investigated progression of knee OA in contrast to the presence of clinical knee OA in the current study. These are two distinct endpoints in the etiology of knee OA, accompanied by distinct risk factors [34], which may also explain contradictory findings for cheese consumption between the OAI and the current study.

Several methodological explanations for the observed inverse association of Dutch cheese and full fat dairy products with knee OA can be put forward. A first methodological question is: could this association be an artefact due to residual confounding or reverse causation? Nearly 80% of the participants defined as having knee OA were overweight or obese. Obesity is the best known risk factor for knee OA development [39], and diet therapy focused at weight reduction by means of caloric restriction including limited consumption of dietary fat is indicated for knee OA patients that are overweight or obese [40, 41]. Consequently, it could be argued that as a result of dietary therapy, consumption of full-fat dairy, including Dutch cheese, was lower in individuals with than without knee OA (as can be seen from Table 3), which would indicate reverse causation. Second, two Dutch studies showed that only 11% of the overweight and 30% of the obese patients in orthopaedic practice received dietary therapy [41], and only 14% of the overweight and obese patients in general practice [42]. Together with the assumption that many individuals with clinical knee OA in our study will not have received an official diagnosis for knee OA (yet), that compliance with dietary advice for weight management is generally poor, BMI did not modify the associations (*P* interaction > 0.10) and results remained significant after correction for BMI, it is unlikely that the observed association between knee OA and Dutch cheese in our study is caused by reverse causation.

From a mechanistic point of view, cheese and especially hard and semi-hard cheese, such as most nutrient-dense Dutch cheese, substantially contribute to the intake of micronutrients important for bone health, such as calcium, phosphorus, magnesium and zinc [43, 44]. However, additional adjustment of the analyses for these micronutrients did not change the results (data not shown), indicating that increased intake of these micronutrients did not mediate the association. Cheese is also one of the most important sources of menaquinones (vitamin K2) in the Dutch food supply [45]; unfortunately, we were unable to evaluate the role of menaquinones because at present the 2011 Dutch Food Composition Database (NEVO) contains insufficient data for reliable estimation of menaquinone intake. During microbial fermentation of milk to cheese, in addition to vitamin K, multiple bioactive agents including bioactive peptides with anti-oxidative properties, are released into the food matrix [46]. These could play a role in the prevention of knee OA; however, no human data are available so far. Finally, since dietary lipids affect bioavailability of fat-soluble nutrients such as vitamin A, D, E, K, carotenoids and phytosterols [47], consumption of high fat foods, including cheese and full fat dairy, can enhance uptake of fat-soluble nutrients from other foods that are consumed simultaneously, which may explain the observed inverse association of Dutch cheese and full fat dairy with knee OA.

Secondary analyses on effect modification revealed a significant inverse relationship between intake of full-fat dairy or Dutch cheese with knee OA in individuals with present or past occupational exposure to knee loading but not for those without. Moreover, for this group exposed to occupational knee loading, significant inverse relationships between total dairy, fermented dairy, and total cheese with knee OA were observed as well. The reason for this remains speculative, but it can be hypothesized that, similar to the synergistic effect of exercise and nutrition on muscle protein synthesis [48], a certain minimum level of repetitive mechanical knee loading together with sufficient dairy intake is necessary to protect the knee joint from structural changes that lead to knee OA.

Major strengths of this study include the large sample size and the extensive data collection that enabled thorough adjustment for dietary factors and known determinants of knee OA. Furthermore, the FFQ used in The Maastricht Study is one of the most comprehensive FFQs used so far in any study, and with 47 items on dairy intake, we believe dairy consumption was estimated optimally. Also, given the scarcity of literature on the role of diet in knee OA physiopathology, our results add valuable new information to elucidate the effect of modifiable lifestyle factors on knee OA. Because many reports on the relation between dietary factors and knee OA are based on American study populations, such as the Framingham knee OA Cohort Study and the Osteoarthritis Initiative, questions can be raised about generalizability of their results to other countries with different typical diets. Complementary information from other countries is, therefore, highly valuable. The results of the present report, based on a Dutch study population, are not in line with previous research in an American [18] and Turkish [17] study population on the relationship between dairy consumption and knee OA, which indeed suggest that between-country differences in type and quantity of dairy consumed may result in different associations between dairy intake and knee OA. Last, unlike earlier research, we examined potential effect modification by occupational knee-loading and found that other lifestyle factors, such as repetitive mechanical strain, may play a modifying role.

Our study also has some limitations. First, as with any cross-sectional design, a major limitation is that causal inference cannot be made. Second, in the absence of data on the clinical sign crepitus in The Maastricht Study, we used a modified version of the ACR criteria which may have altered the discriminative value. Notwithstanding, individuals with knee pain and ≥ 3 out of 5 clinical signs, i.e. excluding crepitus, would have shown ≥ 3 out of 6 clinical signs, as well. To prevent information bias, individuals with knee pain that did not meet our modified ACR criteria were excluded from the analyses and consequently individuals without knee pain served as reference group. This reference group cannot be defined as having knee OA because the presence of knee pain in the main criterion of the ACR clinical classification criteria for OA of the knee. Peat et al. [49] reported that, relative to radiographic grading of OA, the ACR clinical criteria seemed more reflective of advanced rather than early or mild osteoarthritis. When studying associations between dietary factors and prevalent knee OA, we do not consider this to be a true limitation.

In conclusion, in this Dutch population, higher intake of full-fat dairy and Dutch cheese was cross-sectionally associated with the lower presence of clinical knee OA. Previous observations that high milk consumption was associated with lower risk of knee OA [17, 18], was not confirmed in the present study. Prospective studies will be needed to assess the relationship between dairy consumption, and in particular semi-hard cheeses such as Dutch cheese, with incident knee OA in individuals with and without occupation-related squatting/kneeling and heavy lifting.
